# Soil productivity and structure of bacterial and fungal communities in unfertilized arable soil

**DOI:** 10.1371/journal.pone.0204085

**Published:** 2018-09-24

**Authors:** Boxi Wang, Yoichi Adachi, Shuichi Sugiyama

**Affiliations:** 1 Faculty of Agriculture and Life Science, Hirosaki University, Hirosaki, Aomori, Japan; 2 The United Graduate School of Agricultural Sciences, Iwate University, Morioka, Iwate, Japan; Free University of Bozen/Bolzano, ITALY

## Abstract

Soil productivity is strongly influenced by the activities of microbial communities. However, it is not well understood how community structure, including its richness, mass, and composition, influences soil functions. We investigated the relationships between soil productivity and microbial communities in unfertilized arable soils extending over 1000 km in eastern Japan. Soil properties, including C turnover rate, N mineralization rate, microbial C, and various soil chemical properties, were measured. Soil bacterial and fungal communities were analyzed by Illumina’s MiSeq using 16S rRNA and ITS regions. In addition, root microbial communities from maize grown in each soil were also investigated. Soil bacterial communities shared many operational taxonomic units (OTUs) among farms. An ordination plot based on correspondence analysis revealed convergent distribution of soil bacterial communities across the farms, which seemed to be a result of similar agricultural management practices. Although fungal communities showed lower richness and a lower proportion of shared OTUs than bacterial communities, community structure between the farms tended to be convergent. On the other hand, root communities had lower richness and a higher abundance of specific taxa than the soil communities. Two soil functions, decomposition activity and soil productivity, were extracted by principal component analysis (PCA) based on eight soil properties. Soil productivity correlated with N mineralization rate, P_2_O_5_, and maize growth, but not with decomposition activity, which is characterized by C turnover rate, soil organic C, and microbial mass. Soil productivity showed a significant association with community composition, but not with richness and mass of soil microbial communities. Soil productivity also correlated with the abundance of several specific taxa, both in bacteria and fungi. Root communities did not show any clear correlations with soil productivity. These results demonstrate that community composition and abundance of soil microbial communities play important roles in determining soil productivity.

## Introduction

Soil harbors diverse microbial communities. The diversity and composition of these communities vary largely between different environments. For example, the structure of microbial communities can be influenced by soil pH [1–6], soil type [7,8], electrical conductivity (EC) [2,9], nutrient availability [10], climate [11,12], and vegetation type [13]. On the other hand, microbial communities play an important role in determining soil functions such as carbon (C) turnover rate, mineralization of nitrogen (N), and pest control [14–16]. As soil properties influence the microbial community, and the microbial community, in turn, shapes various soil functions, there exists a close association between the soil and the microbes.

Soil productivity is strongly influenced by soil microbial communities. Since crop yield in conventional agriculture depends strongly on the utilization of synthetic fertilizers and various kinds of pesticides, the effects of soil productivity on crop yield have been neglected. In contrast, organic farming does not depend on synthetic fertilizers, and thus crop yield essentially relies on soil productivity. While soil microbes contribute to soil productivity through amelioration of soil physical structure and activation of mineral cycling in soil [16], it is not completely clear how the microbial community influences soil productivity. As the structure of soil microbial communities is heavily altered by the quality and quantity of synthetic fertilizers and manures [17,18], arable lands that have not been exposed to any fertilizers are suitable for investigating the relationships between microbial communities and soil productivity.

The structure of a microbial community is characterized by its mass, abundance, and richness. Chloroform fumigation and the use of phospholipid fatty acids are effective ways to estimate microbial mass, while molecular fingerprinting methods such as denaturing gradient gel electrophoresis (DGGE) and terminal restriction fragment length polymorphism (T-RFLP) have been used to analyze community composition [19]. However, these methods have a limited ability to dissect the structure of microbial communities. Furthermore, despite the fact that fungi are important members of the soil microbial community, much more attention has been paid to bacteria [20,21]. The recent development of next generation sequencing (NGS) technology has enabled a deeper resolution of community structure and identification of a large number of low-abundance taxa in bacterial and fungal communities [22]. In this study, we examined the richness, composition, and abundance of soil bacterial and fungal communities from 12 unfertilized farms extending over 1000 km, where microbial communities have not been exposed to any fertilizers, including manures, for at least the last five years.

We first compared the diversity patterns of soil bacterial and fungal communities across the 12 farms. We investigated how spatial variations in bacterial and fungal communities, including root communities, are organized in organic farms. Second, we examined how soil productivity is affected by microbial communities. Soil productivity is determined by many factors including soil physical and chemical properties, microbial activities, and their interactions. To clarify which soil properties are responsible for soil productivity, we measured C turnover rate when incubated with grass litter, N mineralization rate, and maize growth in each soil, as well as chemical properties such as soil pH, N, phosphorous (P_2_O_5_), and organic C (Co) contents. Our aim was to clarify which characteristics of microbial communities are responsible for soil productivity and its components. Three specific questions were addressed. Among mass, richness, and composition of communities, which components are involved in soil productivity? Are bacterial or fungal communities more important in determining soil productivity? What is the contribution of root communities to soil productivity?

## Materials and methods

### Soil collection

**Fig 1:**
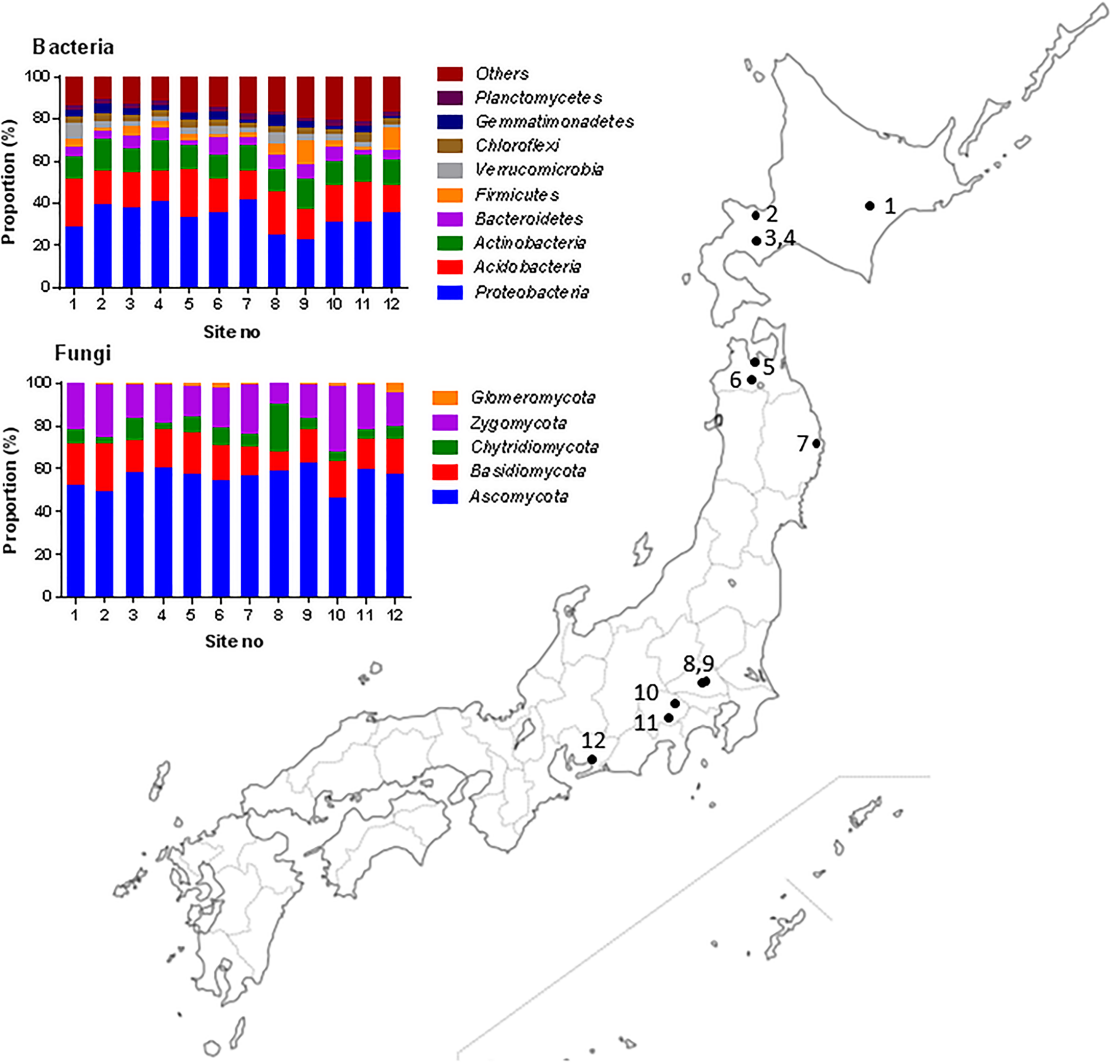
Map of the 12 organic farms used in this study and the composition of bacterial and fungal communities at the phylum level in each farm.

Soils were collected from 12 unfertilized organic farms in eastern Japan with the permission of landowners (Fig 1 and Table 1). The soils were sampled from the 0–10 cm layer at three different locations (replicates) in each site during April and May 2015. After sieving through a 2 mm mesh to remove roots, macrofauna, and rocks, the soils were subjected to chemical and microbial analysis for assessing the growth of maize, and to DNA extraction for microbial community analysis. The soils were stored at 4 °C for the chemical analysis and at -25 °C for DNA analysis until processing.

**Table 1:** Geographic positions and soil properties in each farm.

Site no	Site code	Latitude	Longitude	Soil ^#1^ type	Soil pH	Mineralized N (mg 100g soil^-1^)	P_2_O_5_ (mg 100g soil^-1^)	Soil Organic C (%)	Soil C/N	Microbe C (mg 100g soil^-1^)	C turnover rate (μmol CO_2_ m^-2^ h^-1^)	Maize DW (g kg soil^-1^)
1	OR	43.16	140.78	V	5.6	0.8	8.5	2.78	14.12	27.93	1.08	1.61
2	ST	42.87	143.36	F	5.3	4.1	30.1	3.76	12.59	56.86	1.21	4.68
3	M4	42.62	140.79	V	5.9	1.2	12.5	1.31	9.23	28.50	1.16	2.27
4	M9	42.62	140.79	V	6.2	1.5	18.7	2.16	11.64	38.32	1.18	2.91
5	H	40.85	140.67	A	4.7	2	5.1	2.19	10.51	45.66	1.08	2.29
6	N	40.55	140.55	A	6	1.6	23.5	1.80	8.00	36.52	1.23	2.46
7	IZ	39.32	141.45	F	5.9	3.9	29.9	0.91	8.35	28.25	1.47	3.93
8	SE	35.84	139.54	V	5.8	0.1	5	4.23	12.26	61.46	2.53	1.73
9	SI	35.85	139.56	A	6.2	1.5	14.4	2.28	10.28	33.99	1.31	2.53
10	OG	35.9	138.53	V	6.7	4.6	34.5	4.63	11.48	68.87	1.91	3.73
11	Y	35.86	138.39	V	5.8	4.7	1.3	5.78	10.61	97.08	1.95	3.47
12	IM	34.8	137.01	A	6.4	1.4	111.4	1.57	9.55	39.14	1.19	2.53
Significance					***	***	***	***	***	***	***	***
LSD ^#2^					0.47	1.3	8.1	0.57	2.42	32.67	1.06	1.19

***: significance at 0.1%

^#1^: Soil type; V: volcanic ash soil, F: brown forest soil, A: alluvial soil

^#2^: Least significant difference (LSD) based on Tukey-Kramer HSD test.

### Chemical and microbial analysis

Available N (NO_3_^-^ and NH_4_^+^) and P were measured via colorimetric assay with a spectrophotometer (Jasco V-630 BIO, Tokyo, Japan). After extraction of 5 g dry soil in 50 mL of 2 M KCl solution, NO_3_^-^ and NH_4_^+^ were determined using the sodium salicylic acid and indophenol blue methods, respectively [23]. The available P_2_O_5_ content was determined using the ammonium molybdate-ascorbic acid method, following the extraction of 5 g dry soil in a 50 mL solution of 0.05 M ammonium sulfate [24]. Soil total N content (%) and total C content (%) were determined using an automatic N-C analyzer (Vario EL cube. Elementar, Langenselbold, Germany). The soil pH was determined with a pH meter (Horiba pH Meter D-52, Tokyo, Japan) after dissolving 10 g of dry soil in 25 ml of distilled water.

In addition, three microbial traits, C turnover rate, N mineralization rate, and microbial mass, were measured using fresh soil. C turnover rate was evaluated for the soil incubated with leaf litter from C_3_ grass *Dactylis glomerata*, which has high nitrogen content and a high decomposition rate [25]. A polyvinyl plastic pot (19.6 cm^2^ area and 3.5 cm height) was filled with 25.0 g fresh soil along with dried leaf litter (0.3 g). Sterilized distilled water was added to all the soils to standardize the moisture content to 60%. The samples were incubated at 25 °C for 40 days. The CO_2_ efflux rate was monitored using an automatic CO_2_ analyzer (Li-6400; Li-Cor Biosciences, Lincoln, NE, USA) equipped with a soil CO_2_ emission chamber (LI-6400–09; Li-Cor). The CO_2_ concentration inside the chamber was set at 400 μmol during the measurements. The changes in the CO_2_ concentration were monitored for 60 s, and the CO_2_ efflux from the soil was calculated in μmol CO_2_ m^–2^ s^–1^. As the CO_2_ efflux rate reached a peak value at 3 days after the incubation and then rapidly declined, the maximum rate at 3 days was used as the C turnover rate. The N mineralization rate was measured by incubating the soil for 40 days without the addition of grass litter. The available N was measured before and after the incubation, and N mineralization rate was calculated as the difference in available N content during the incubation period (40 days). Microbial mass was measured using the chloroform fumigation method [26]. Fresh soil (8 g) was placed into a 100 mL glass beaker and fumigated with 50 mL chloroform under reduced pressure in a vacuum desiccator for 24 h. After extracting 50 mL of 0.5 M potassium sulfate solution, soil C was measured with an automatic carbon analyzer (Shimadzu TOC-L, Kyoto Japan). Soil microbial C was calculated as the difference between the control and the fumigation treatment multiplied by a correction factor (2.64).

Soil productivity was evaluated using the dry mass of maize grown on each soil. Maize seeds were sown on a wet filter paper for germination, and on July 7, three seedlings were transplanted into pots (113 mm diameter and 184 mm depth) containing each of the soils and grown in a greenhouse. Three pots (replicates) for each soil were prepared. After 20 days, the shoot and root of the maize were harvested. The root parts were washed to remove attached soil, and 2 g of fresh root tip was taken and stored at -25 °C for DNA extraction. After drying at 70 °C for 48 h, the root and shoot dry weights were measured. As there was a large difference in the bulk density among soils, maize weight was expressed as per g of dry soil in a pot, not as per pot.

### DNA extraction

As DNA extraction using commercial extraction kits was difficult for some soil samples from volcanic ash, the DNA was extracted by bead-beating and CTAB-based method of Hoshino [27] with slight modifications. This method can be applied for DNA extraction from roots. Soil samples (400 mg) were smashed with 0.1 mm glass beads, 800 μL extraction buffer (0.1 M NaCl, 0.1 M Tris–HCl, 0.1 M EDTA, and 0.3 M sodium phosphate), 2% SDS, and 3.2 mg skim milk at 5500 rpm for 40 s. The samples were then subjected to centrifugation at 18,000 ×*g* for 1 min, and the supernatants were transferred to a new tube and incubated for 5 min with 400 μL of 5% CTAB, 30 μL of 5 M potassium acetate, and 550 μL of chloroform-isoamyl alcohol (24:1). After centrifugation at 10,000 ×*g* for 5 min, the upper phase was transferred to a new tube and incubated at 55 °C for 1 h after mixing it with 800 μL of CTAB precipitation solution (5 g L^-1^ CTAB, 0.04 M NaCl). The samples were centrifuged for 5 min at 18,000 ×*g*, following which, the supernatant was discarded and the pellet was washed with 500 μL of ethanol (70% v/v). The pellet was dried for 1 h, and the DNA was dissolved in 50 μL of sterile deionized water.

The 100 mg root sample was smashed with a 0.5 mm zirconia bead, 0.2 mm glass beads, and 100 μL of extraction buffer (0.5 M NaCl, 0.1 M Tris, 0.1 M EDTA), following which, it was incubated with 700 μL of 1.3% CTAB, 20 μL of 2-mercaptoethanol, and 700 μL of chloroform-isoamyl alcohol (24:1). After precipitation and purification by the same method mentioned above, DNA was dissolved in 50 μL of sterile deionized water.

### PCR amplification and sequencing

For bacteria, a V4 region of 16S ribosomal RNA (rRNA) was used [28]. The primer pair in the 1st PCR amplification was 515F (5′—GTGCCAGCMGCCGCGGTAA—3′) and 816R (5′—GGACTACHVGGGTWTCTAAT—3′) with adaptors. To reduce the chloroplast and mitochondrial amplification, 2.5 pmol μL^-1^ peptide nucleic acid (PNA) clamps were included in the reaction according to Lundberg [28]. For fungi, the ITS2 region of rRNA was used [29]. The primer pair for the 1st PCR was fITS (5′—GTGARTCATCGAATCTTTG—3′) and ITS4 (5′—TCCTSCGCTTATTGATATGC—3′) with adaptors. The PCR conditions were as follows: an initial denaturation at 94 °C for 30 s, and 25 cycles at 94 °C for 15 s, 50 °C for 30 s, and 72 °C for 30 s, with a final extension at 72 °C for 5 min. After purification by AMPure XP magnetic beads (Beckman-Coulter, Indianapolis, IN, USA), the 1st PCR products were followed by the second PCR with the primer pair, 2nd-F (AATGATACGGCGACCACCGAGATCTACAC -Index2 -ACACTCTTTCCCTACACGACGC) and 2nd-R (CAAGCAGAAGACGGCATACGAGAT—Index1 -GTGACTGGAGTTCAGACGTGTG), for both 16S rRNA and fungal ITS2. The index pair was specific to each sample, for an accurate recognition of the samples. The second PCR conditions were: 94 °C for 2 min, 8 cycles at 94 °C for 10 s, 60 °C for 30 s, 72 °C for 30 s, with a final extension at 72 °C for 5 min. The second PCR products were purified using the AMPure XP magnetic beads and pooled in equimolar ratios. After confirming the library quantity, paired-end 2 × 250 bp sequencing of the barcoded amplicons was performed on a MiSeq machine (Illumina Inc, San Diego, CA, USA).

### Sequence processing

The sequences obtained from the MiSeq were processed through a custom pipeline developed at Fasmac Inc. (Atsugi, Japan). The raw reads were demultiplexed based on the barcode sequences and filtered by exact matching using Fastx toolkit (fastq_barcode_splitter). If the quality score was less than 20 and the sequence length was less than 40 bases, the reads were discarded. Paired-end reads with a minimum 10 base overlap were merged into full-length sequences by FLASH v1.2.10 [30]. Merged sequences between 246 and 260 bases were used for later processing of 16S rRNA. Chimeric sequences were detected using the UCHIME algorithm from the USEARCH package [31]. Operational taxonomic unit (OTU) generation and phylogenetic assignment were conducted using QIIME (v1.9.0) script with default conditions. OTUs were clustered using UCLUST [32] at a 97% similarity level by a de novo picking method, using Greengenes 16S reference database [33] for 16S V4 data and UNITE reference database [34] for ITS2 data. To obtain the filtered OTU dataset, all OTUs assigned to Archaea, chloroplast, and mitochondria were discarded from the 16S dataset, and only the OTUs assigned to kingdom Fungi were reserved for the ITS2 dataset. The filtered datasets were then normalized by transforming the number of OTU counts to relative abundance values. The raw reads of sequences were deposited into the DDBJ Sequence Read Archive (DRA) database (DRA accession: DRA006580).

### Statistical analysis and visualization

The differences in soil properties between the farms were tested with ANOVA and Tukey-Kramer HSD for multiple comparisons. Since the residual variations for all traits were distributed randomly and were independent from the mean values, no transformations of the data for the improvement of normality and homoscedasticity were applied. Since soil properties were highly correlated with each other, a principal component analysis (PCA) based on the correlation matrix was applied to extract independent ordination axis. These statistical analyses were conducted using JMP (v4.0, SAS, Cary NC, USA).

As spurious sequences and unrepresentative OTUs decrease the reproducibility for community assemblage [35], the generated OTUs were filtered at the threshold of 0.01% of the total abundance, and the OTUs above this level were used for the diversity analysis. Community richness was evaluated using the number of OTUs. Although various measures evaluating α- and β-diversity have been proposed, the Shannon-Weaver index (H′) was used in this study, as this method enables the total diversity (γ diversity) to be partitioned into independent α- (within-site) and β-diversity [36].

$$H\prime =\;-\;\Sigma \;\left({\text{N}}_{\text{i}}{\text{/N}}_{\text{t}}\right)\;*\;\text{ln}\;\left({\text{N}}_{\text{i}}{\text{/N}}_{\text{t}}\right)\text{,}$$

where N_i_ is the number of reads in each OTU and N_t_ is the sum of OTUs. To avoid N_i_ = 0 during the calculation of logarithm, 1 was added for all N_i_.

To detect any differences in relative abundance between soil and root communities, a paired *t*-test was applied for each taxon. The community structure was evaluated by canonical correspondence analysis (CCA), based on the relative abundance of each taxon. As the taxonomic identification of OTUs was not possible below the order level for bacteria, OTUs belonging to each taxon were summed at the order level, and their relative abundances were calculated for both bacteria and fungi. The relative abundance for each taxon was also calculated at the class and phylum level. A permutation ANOVA was used to examine the relationships between community ordination structure by CCA and soil properties. CCA analysis was conducted using the VEGAN package in R (v3.1.1) [37].

## Results

### Microbial community structure

The total diversity across all sites (γ-diversity) was partitioned into α- and β-diversity using the Shannon-Weaver index (Table 2). The α-diversity was twice as high as the β-diversity in bacterial and fungal communities (Table 2 and S1 Fig), and thus the total diversity was mostly ascribed to within-farm variation. The soil community had a significantly higher α-diversity than the root community both for bacteria and fungi (p < 0.01, *t*-test). The bacteria showed a significantly higher α-diversity than fungi in both the soil and the root communities (p < 0.01, *t*-test). The total number of OTUs in soil was 5337 for bacteria and 2569 for fungi. The total OTU richness in the root community was much lower than in the soil community (3791 for bacteria and 1082 for fungi). The mean number of OTUs in the soil community for single sites was 2785 ± 90 for bacteria and 498 ± 32 for fungi and in the root community was 1771 ± 89 for bacteria and 251 ± 16 for fungi.

**Table 2:** α-, β-, and γ-diversity, and the number of observed OTUs for soil and root microbial communities. The α-, β- and γ-diversity were calculated at the OTU level.

	Bacteria		Fungi	
	Soil	Root	Soil	Root
α-diversity	5.99	4.54	4.25	2.86
β-diversity	2.48	2.48	2.48	2.10
γ-diversity	8.64	7.02	6.80	4.96
No. of observed OTUs	5337	3791	2569	1082

**Fig 2:**
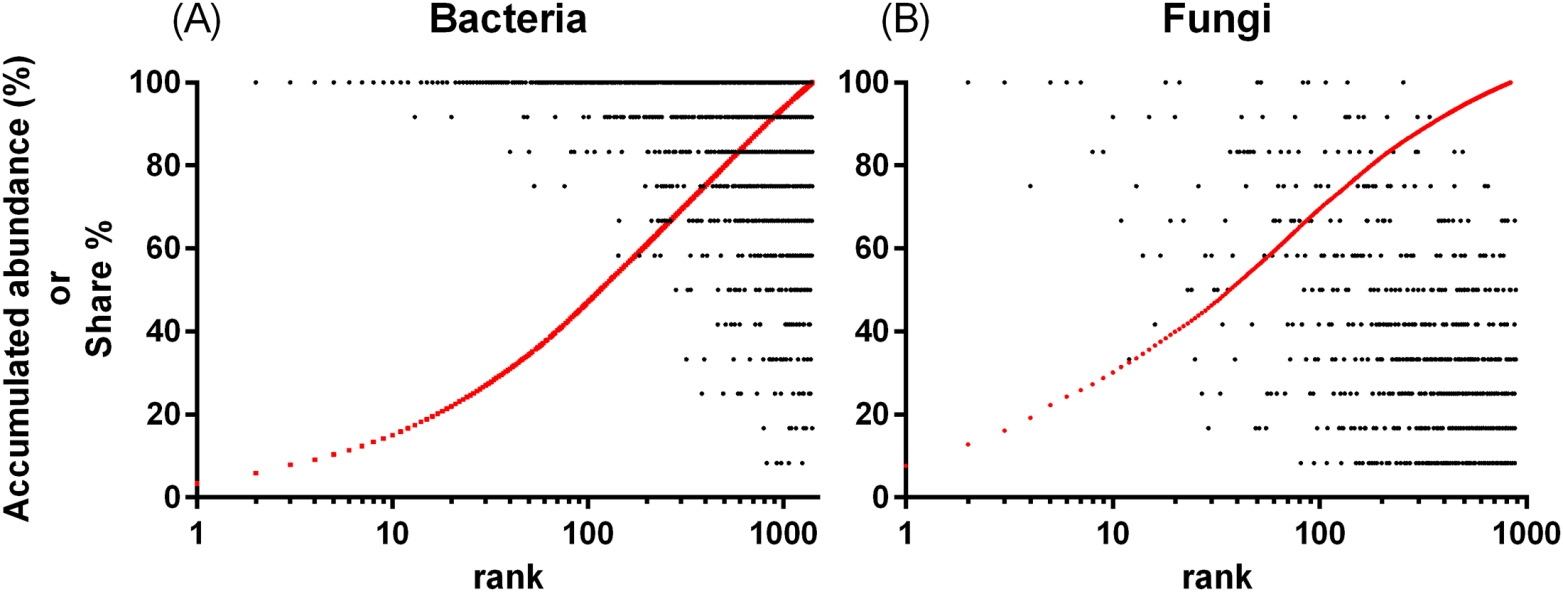
Abundance structure and overlap of each OTU in soil bacterial and fungal communities from 12 farms. The proportion of OTUS shared among the 12 farms (black) and the accumulated proportion (red) are shown according to the rank order of each OTU.

In spite of the high richness, the total abundance was largely comprised of the highest-ranked OTUs; the top 100 OTUs accounted for 47.1% of the total abundance for bacteria and 61.9% for fungi, as shown in Fig 2. The high-ranked OTUs tended to be shared by a greater number of sites than the low-ranked OTUs, in both bacterial and fungal communities.

**Fig 3:**
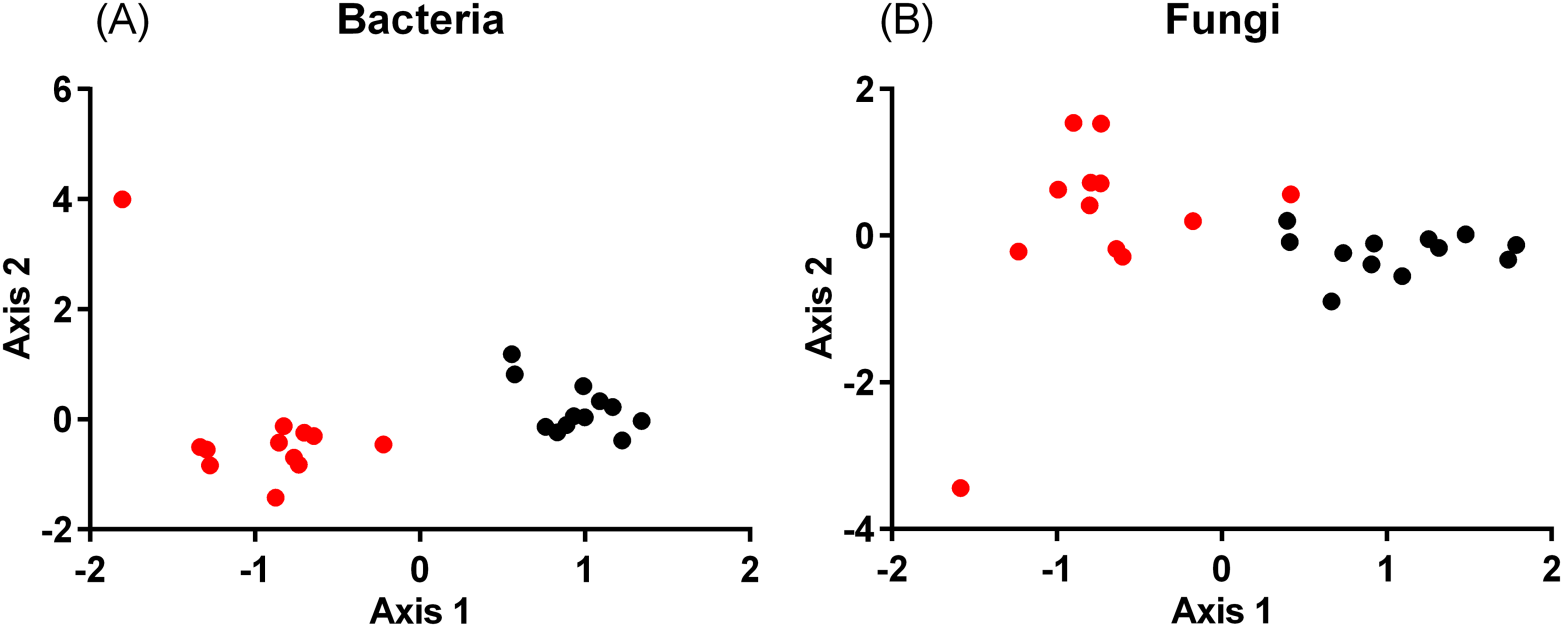
Ordination plots of correspondence analysis based on the relative abundance at the order level in soil (black) and root (red) bacterial and fungal communities.

The correspondence analysis based on the relative abundance revealed a convergent distribution of soil bacterial communities in an ordination plot (Fig 3). Although the fungal communities had a lower proportion of the OTUs shared among all sites (1.8%) than the bacterial communities (31%), we still obtained community convergence in the ordination plot (Fig 3).

**Fig 4:**
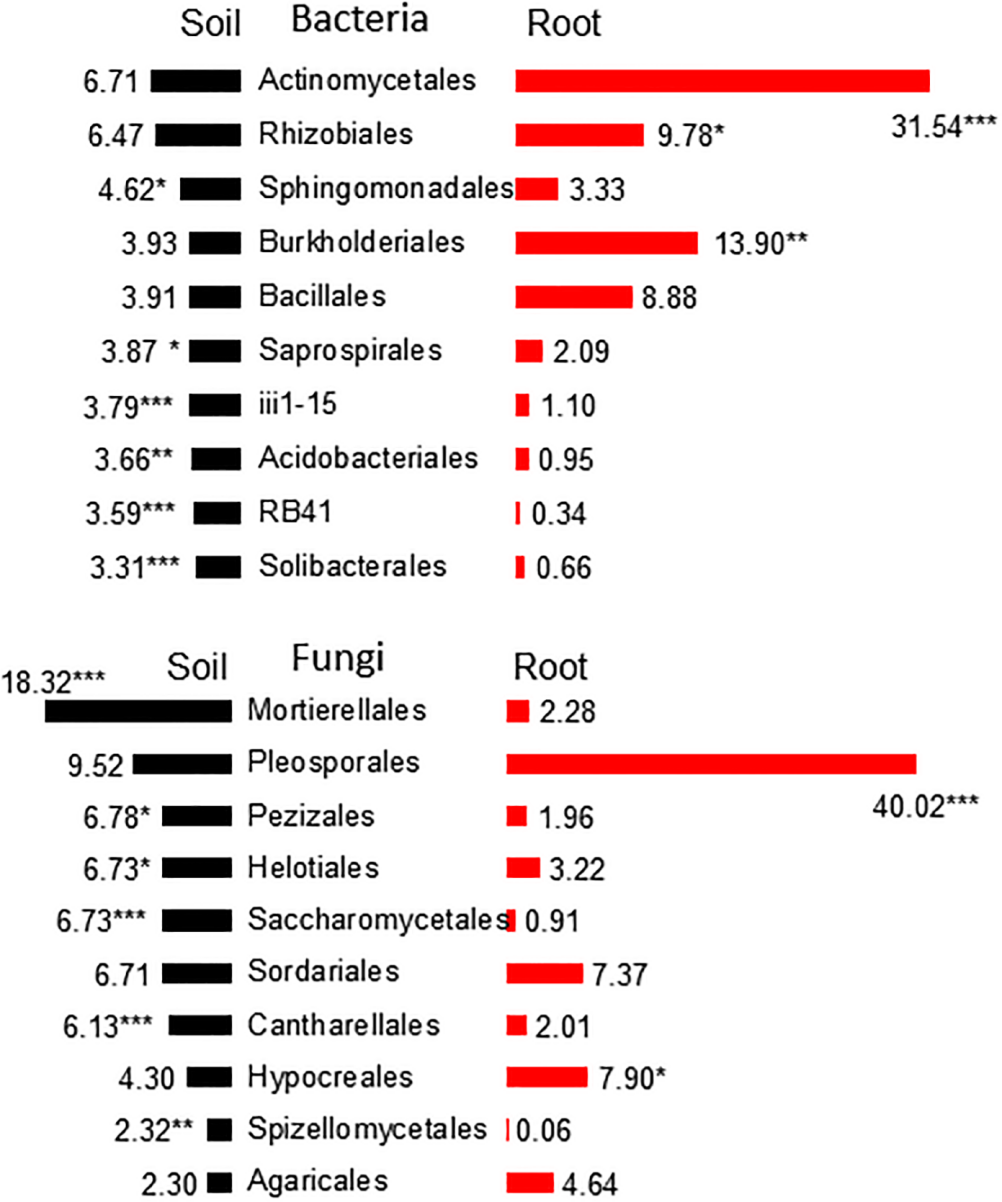
The relative abundance (%) of the top 10 taxa at the order level in soil (black) and root (red) bacterial and fungal communities. Significant differences between the root and soil communities were detected by a paired *t*-test (* p < 0.05; ** p < 0.01; *** p < 0.001).

Three phyla, *Proteobacteria*, *Acidobacteria*, and *Actinobacteria*, comprised more than 60% of the total abundance in the soil bacterial communities (Fig 1 and S1 Table). At the order level, the mean abundance of the three dominant groups, *Actinomycetales*, *Rhizobiales* and *Sphingomonadales*, were 6.7% ±0.47, 6.5% ± 0.62 and 4.6% ± 0.61, respectively, as shown in Fig 4. More than 80% of the abundance in soil fungal communities was due to three phyla (*Ascomycota*, *Zygomycota*, and *Basidiomycota*), while the order *Mortierellales* accounted for 18.3% ±1.51(se) of the abundance (S2 Table).

A smaller number of high-ranked OTUs showed higher abundance in the root communities than in the soil. Three bacterial orders, *Actinomycetales*, *Rhizobiales*, and *Burkholderiales*, were significantly enriched at the root (Fig 4 and S3 Table). Notably, *Actinomycetales* increased its abundance by 31.5% (Fig 4), whereas six other orders showed a significantly lower abundance. As for the fungi, orders *Pleosporales* and *Hypocreales* were significantly enriched at the root, with the order *Pleosporales* showing an especially high enrichment of 40.0% (Fig 4).

### Soil productivity and microbial communities

**Fig 5:**
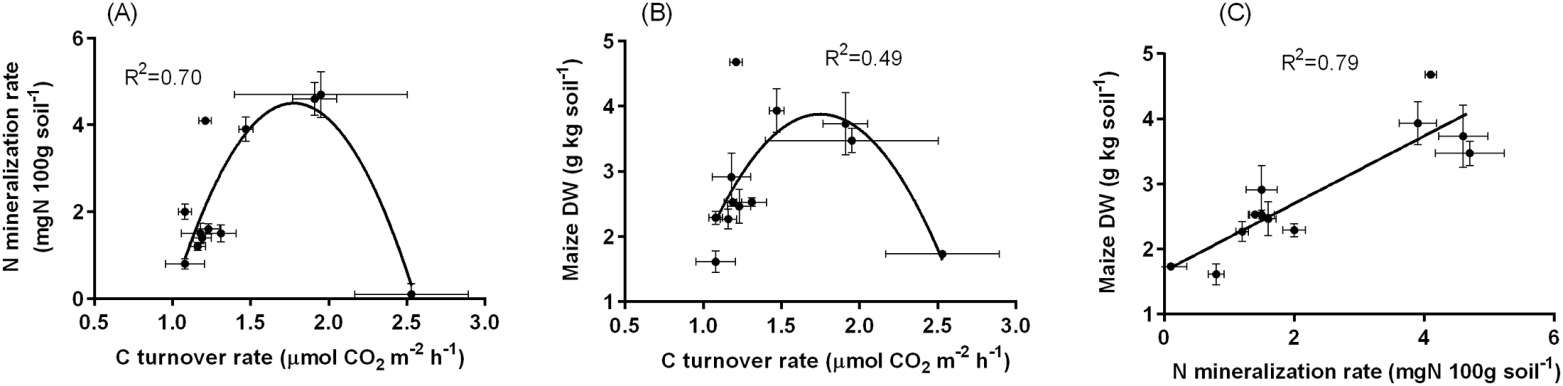
The relationships between C turnover rate, N mineralization rate, and maize dry matter weight (DW) in soils from 12 farms.

The eight soil measurements [soil pH, mineralized N (Nm), P_2_O_5_, soil organic C (Co), C/ N ratio, microbe C (Cm), C turnover rate (R), and maize dry weight (DW)] showed significant differences at the 0.1% level across the 12 sites (Table 1). Pearson correlation analysis (Fig 5 and S5 Table) showed that R was significantly positively correlated with Co (r = 0.68*) and Cm (r = 0.67*). In contrast, the maize DW linearly increased with mineralized N (r = 0.89***) but not with R (r = 0.03). These correlation patterns indicated that the two microbial processes, C turnover and N mineralization, were not correlated with each other (Fig 5). The principal component analysis (PCA) discriminated between these two functions (Table 3). The first component (PC1), which explained 39.6% of the total variation, was characterized by Co, Cm, and R, and thus represents microbial decomposition activity, while the second component (PC2), which explained 24.3% variation, was characterized by maize DW, P_2_O_5_, and Nm, and thus, represents soil productivity.

**Table 3:** Normalized eigen vectors for eight soil properties and the proportion of variation explained (%) by the three PCA components (PC1, PC2, and PC3). The eight soil properties were: soil pH, mineralized N (Nm), P_2_O_5_ content, soil organic carbon content (Co), soil C/N ratio, microbial biomass (Cm), C turnover rate (R), and maize dry weight (DW).

	PC1	PC2	PC3
Eigen vectors			
pH	0.04	0.52	-0.83
Nm	0.73	0.65	0.4
P_2_O_5_	-0.3	0.7	-0.41
Co	1.02	-0.27	-0.13
C/N	0.38	-0.65	0.05
Cm	1.02	-0.02	-0.09
R	0.75	-0.22	-0.56
DW	0.56	0.75	0.44
%	39.6	24.3	16.6

**Fig 6:**
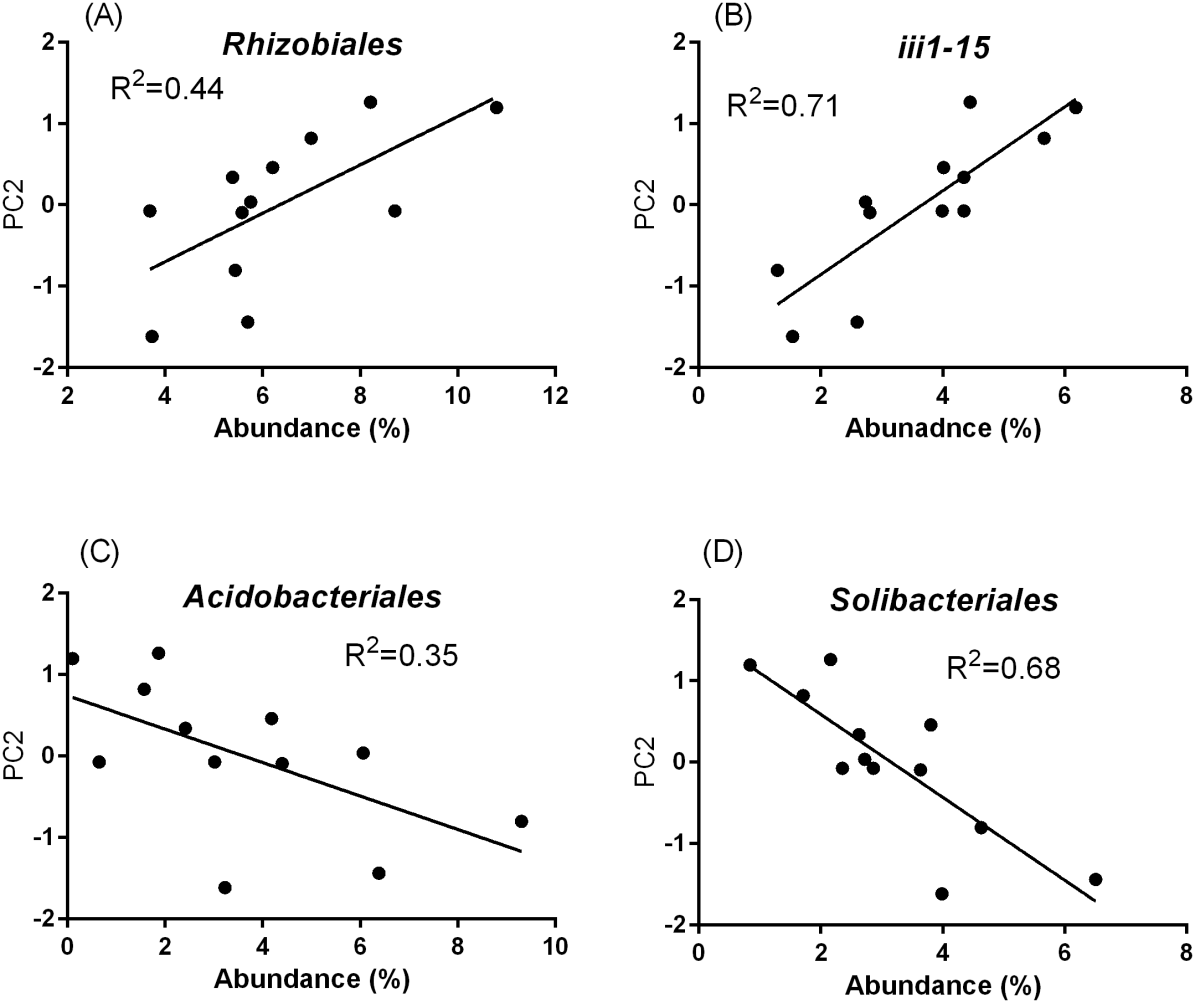
The relationships between soil productivity (PC2) and the relative abundance of the four orders (*Rhizobiales*, *iii1-15*, *Acidobacteriales*, and *Solibacterales*) in soil bacterial communities.

Richness did not show any significant correlations with decomposition activity (PC1) or soil productivity (PC2) in bacterial or fungal communities. On the other hand, the mass of soil microbes was closely associated with decomposition rate (PC1) but not with soil productivity (PC2). Canonical correspondence analysis (CCA) revealed that soil productivity (PC2) was strongly influenced by the community structure at the order level in both bacteria and fungi (Table 4). However, significant effects of community structure were not found at the higher taxonomic levels of class and phylum. The relationships between the abundances of the top 10 taxa at the order level and soil productivity were examined (Fig 6). Soil productivity (PC2) was positively correlated with the relative abundances of *Rhizobiales* (r = 0.651*) and *iii1-15* (r = 0.827***), and negatively with *Acidobacteriales* (r = -0.606*) and *Solibacterales* (p = -0.839***). For fungi, only *Pezizales* (r = -0.724**) showed a significant correlation with soil productivity (S6 Table).

**Table 4:** Results of a permutation ANOVA examining the effect of three PCA components (Table 3) and the latitude of farms on the structure of soil bacterial and fungal communities. The evaluation was done by correspondence analysis based on the abundances at three taxonomic levels: phylum, class, and order.

	Bacteria			Fungi		
	Phylum	Class	Order	Phylum	Class	Order
PC1	0.80	0.68	0.91	0.62	0.92	0.96
PC2	1.26	1.08	2.40*	1.29	0.90	1.85*
PC3	1.17	1.04	2.59**	2.35*	1.69	1.58
Latitude	1.37	1.56	2.00*	1.81	1.17	1.22

The F ratios are shown, along with the significance

* p < 0.05;

** p < 0.01;

*** p < 0.001

On the other hand, bacterial orders *Sphingomonadales* (r = 0.631, p < 0.05) and S*olibacterales* (r = 0.579, p < 0.01) were significantly correlated with the latitude of the sampling site (S6 Table). Soil pH (PC3 in PCA) was also significantly correlated with the bacterial orders, *Saprospirales* (r = -0.595, p < 0.05), *Acidobacteriales* (r = 0.627, p < 0.05), and RB41 (r = -0.753, p < 0.01). These results demonstrate that environmental factors can also have strong effects on the abundance of each taxa, especially in bacterial communities.

## Discussion

### Structure of bacterial and fungal communities

Although detailed analyses of the community structure of soil microbes have been lagging because of a lack of appropriate methods, recent advances in NGS analysis have helped reveal a picture of extremely diverse soil microbial communities [38,39]. In this study, a 0.4 g soil sample included an average of 2785 bacterial OTUs, which belonged to 150 different taxa at the order level. In contrast to this high within-site diversity, the soil bacterial communities shared many OTUs between farms, even those that were 1000 km apart from each other. Another study had also reported that a small proportion of phylotypes comprises almost half of the total abundance of bacterial communities worldwide [39]. Therefore, sharing of common dominant taxa across spatially distant farms seems to be a universal pattern in soil bacterial communities. On the other hand, soil bacterial communities in this study showed a lower β-diversity than α-diversity (Table 2), which indicates the absence of clear community divergence between farms. This result does not agree with other studies, which have reported a high β-diversity and community divergence in soil bacteria [40].

Dispersal limitation and environmental selection are predominant factors that cause community divergence across sites [41]. Dispersal limitation increases the community divergence between geographically distant sites, while selection causes community divergence depending on differences in environmental conditions across sites. Therefore, the spatial patterns of α- and β-diversity indicate the relative importance of external factors in shaping the structure of soil microbial communities. Furthermore, it is known that microbes establish distinctive communities in plant roots [42], which can potentially influence the structure of soil microbial communities. Although the latitude of the sampling sites showed a significant effect on the structure of bacterial communities (S6 Table), the sharing of most OTUs among distant farms suggests that dispersal limitation does not play an important role in the shaping of bacterial communities. On the other hand, based on the abundance, latitude and soil pH had significant effects on the community structure (Tables 3 and 4), and therefore the effects of environmental selection seemed to be prevalent in the bacterial communities in spite of their low β-diversity. In agricultural lands, soil bacterial communities show a large divergence between conventional and organic farms [43,44], between different tillage regimes [45,46], and between nitrogen treatments [47,48]. Therefore, the low β-diversity of bacterial communities seems to be related to similar management conditions at the farms examined in this study, which could cause directional selection and resulting convergence of the communities. Although fungal communities consisted of a smaller number of OTUs (an average of 577) per soil sample and a lower proportion of shared OTUs among the farms than the bacterial communities, α-diversity was still higher than β-diversity and soil communities were still less divergent in the ordination plot (Fig 3), suggesting the importance of cultivation management on the structure of fungal communities.

Maize root had distinctive communities from the bulk soil, for both bacteria and fungi (Fig 3). We observed an increased abundance of the bacterial orders *Actinomycetales*, *Rhizobiales*, and *Burkholderiales* in maize root, which is in accordance with previous studies of rhizosphere soil [20,49,50]. *Actinomycetales* is reported to confer beneficial functions, including plant disease suppression and plant growth promotion, to host plants [51]. *Rhizobiales* contributes to nutrient cycling, production of phytohormones, and plant growth promotion [52,53]. On the other hand, maize root enriched the fungal orders *Pleosporales* and *Hypocreales*. These fungal groups contain a large number of plant-associated pathogens [41,54], which might have negative effects on the host plants [50]. The presence of distinctive microbial communities in plant roots, likely caused by differential ability of various taxa to colonize root tissues, reflects the ecological interactions between host plants and soil microbes.

### Soil productivity and microbial communities

Soil productivity is one of the most important soil functions and is closely associated with microbial structure and activity [16,55,56]. Although soil productivity in agricultural lands is influenced by many factors, the cycling of mineral nutrients such as C, N, and P should play an important role in determining the soil productivity in unfertilized soils [10,16]. Maize DW was not correlated with C turnover rate but was correlated with N mineralization rate, demonstrating the importance of N cycling in determining soil productivity (Fig 5).

Inorganic N in the soil under unfertilized conditions is supplied mainly via organic N mineralization and microbial N fixation. Most inorganic N is released from organic matter in soil during microbial decomposition and a part of the inorganic N is reabsorbed by microbes for their growth [57]. Therefore, active microbial activities may lead to low N mineralization through reabsorption of inorganic N, as shown in Fig 5. Carbon turnover rate and N mineralization rate, both of which are derived from microbial processes, are not necessarily correlated with each other. In fact, a significant correlation between soil respiration rate and soil productivity has been reported in some studies [55,58], but not in others [59].

N fixation is the other source for N supply to soil. N fixation in the soil is performed by free-living bacteria as well as symbiotic bacteria [60]. *Rhizobiales*, which showed a significant positive correlation with soil productivity, contains various members with N-fixation capabilities [39,61,62]. On the other hand, order *Solibacterales*, which showed a negative correlation with soil productivity, is reported to be a potential indicator for soil degradation [63]. Therefore, soil productivity and supply of inorganic N in soil should be strongly affected by the composition of microbial communities, especially by the abundance of microbial groups with low demands for N and N-fixing ability.

Many studies have reported the existence of growth-promoting bacteria in roots. However, high abundance OTUs in root communities did not show any significantly positive correlations with maize DW. Taxa below the order level were not discriminated in this study. The lower taxonomic resolution may clarify the close association between beneficial functions of host plants and specific microbial groups in root communities.

## Conclusion

The present study revealed extremely high diversity and a disproportionate contribution of a small number of phylotypes to the total abundance in soil bacterial and fungal communities. Soil productivity and its components showed close associations with the abundances and composition of communities rather than with diversity *per se*. We identified four orders in bacteria and one order in fungi that showed significant correlations with soil productivity. Since these taxa were shared among all farms, changes in their abundance through agricultural management could contribute to improvements in soil productivity in farms. Although there is still little information about the most effective farm management practices and soil conditions for altering community composition in a way that leads to increased soil productivity, this study could inform further studies on the relationships between microbial communities and soil functions.

## Supporting information

S1 Fig Rarefaction curve of soil bacterial 16s rRNA sequences (A) and soil fungal ITS sequences (B). (DOCX)

S1 Table Relative abundance of soil bacterial taxa at order level in 12 farms. (XLSX)

S2 Table Relative abundance of soil fungal taxa at genus level in 12 farms. (XLSX)

S3 Table Relative abundance of root bacterial taxa at order level in 12 farms. (XLSX)

S4 Table Relative abundance of root fungal taxa at genus level in 12 farms. (XLSX)

S5 Table Correlation coefficients among eight soil properties and OTU richness in soil bacterial communities. (XLSX)

S6 Table The relationships of soil properties and the latitude of farms with the whole community structure and the abundance of the top 10 orders in soil bacterial and fungal communities. (XLSX)
