# Supplementary figures and images for: Soil productivity and structure of bacterial and fungal communities in unfertilized arable soil
Source: PLoS One. 2018 Sep 24;13(9):e0204085. doi: 10.1371/journal.pone.0204085 (PMC6152964; doi:10.1371/journal.pone.0204085)

(A)


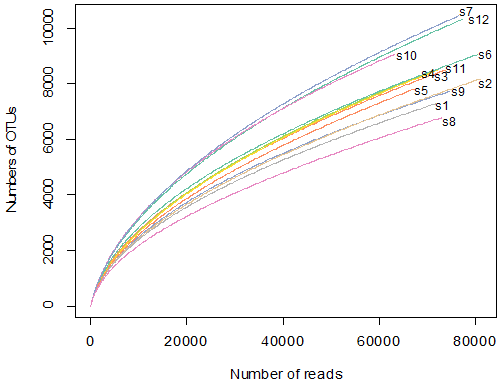


(B)


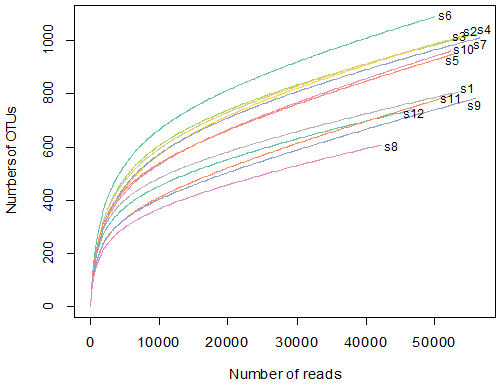


S Fig 1. Rarefaction curve of soil bacterial 16s rRNA sequences (A) and soil fungal ITS sequences (B).

Supplement: S1 Fig — (DOCX) [file pone.0204085.s001.docx]
